# Inactivation of *Escherichia coli* O157:H7 and *Listeria monocytogenes* in biofilms by pulsed ultraviolet light

**DOI:** 10.1186/s13104-015-1206-9

**Published:** 2015-06-10

**Authors:** Nedra L. Montgomery, Pratik Banerjee

**Affiliations:** Department of Food and Animal Sciences, Alabama A&M University, Huntsville, AL 35762 USA; Division of Epidemiology, Biostatistics, and Environmental Health Science, School of Public Health, The University of Memphis, Memphis, TN 38152 USA; General Mills, Inc., Golden Valley, MN USA

**Keywords:** *Escherichia coli* O157:H7, *Listeria monocytogenes*, Biofilms, Pulsed ultraviolet (PUV) light, Microbial inactivation, Lettuce, Low-density polyethylene (LDPE)

## Abstract

**Background:**

The inactivation of biofilms formed by pathogenic bacteria on ready-to-eat and minimally processed fruits and vegetables by nonthermal processing methods is critical to ensure food safety. Pulsed ultraviolet (PUV) light has shown promise in the surface decontamination of liquid, powdered, and solid foods. In this study, the antimicrobial efficacy of PUV light treatment on nascent biofilms formed by *Escherichia coli* O157:H7 and *Listeria monocytogenes* on the surfaces of food packaging materials, such as low-density polyethylene (LDPE), and fresh produce, such as lettuce (*Lactuca sativa*) leaves, was investigated.

**Results:**

The formation of biofilms on Romaine lettuce leaves and LDPE films was confirmed by crystal violet and Alcian blue staining methods. Inactivation of cells in the biofilm was determined by standard plating procedures, and by a luminescence-based bacterial cell viability assay. Upon PUV treatment of 10 s at two different light source to sample distances (4.5 and 8.8 cm), viable cell counts of *L. monocytogenes* and *E. coli* O157:H7 in biofilms on the lettuce surface were reduced by 0.6–2.2 log CFU mL^−1^ and 1.1–3.8 log CFU mL^−1^, respectively. On the LDPE surface, the efficiency of inactivation of biofilm-encased cells was slightly higher. The maximum values for microbial reduction on LDPE were 2.7 log CFU mL^−1^ and 3.9 log CFU mL^−1^ for *L. monocytogenes* and *E. coli* O157:H7, respectively. Increasing the duration of PUV light exposure resulted in a significant (*P* < 0.05) reduction in biofilm formation by both organisms. The results also revealed that PUV treatment was more effective at reducing *E. coli* biofilms compared with *Listeria* biofilms. A moderate increase in temperature (~7–15°C) was observed for both test materials.

**Conclusions:**

PUV is an effective nonthermal intervention method for surface decontamination of *E. coli* O157:H7 and *L. monocytogenes* on fresh produce and packaging materials.

## Background

The contamination and persistence of pathogenic bacteria in certain fresh produce, including ready-to-eat products, have become an emerging concern in recent years. Minimally processed, ready-to-eat fruits and vegetables may contain human pathogens among their microflora owing to contamination at some point in the process from cultivation to consumption. Microbial contamination of fruits and vegetables may occur on the surface or may become internalized through cuts or crevices on the produce [1]. The presence of viable human pathogens in ready-to-eat fresh produce poses a significant food safety risk to consumers. Decontamination of fresh produce presents a challenge for the food processing industry as ready-to-eat fresh produce cannot be treated with heat (thermal processing). Nonthermal processes such as washing with aqueous sanitizer/antimicrobial agents (hypochlorite, peroxyacetic acid, hydrogen peroxide, trisodium phosphate, organic acids) [2, 3], gaseous antimicrobial treatments (ozone, chlorine dioxide) [3, 4], and some physical methods (such as gamma-irradiation) [5] have been employed to reduce the pathogen load on fresh produce. Among these nonthermal processing methods, the application of pulsed ultraviolet (PUV) light or pulsed-light for microbial decontamination of food surfaces, powders and liquid foods is well documented [6–13]. The US Food and Drug Administration approves certain applications of pulsed light for surface microbiological control of food products and food production environments [14, 15]. This method, or variations on it, are also approved for the microbial inactivation of food contact surfaces, packaging materials, and medical devices in the European Union, Canada, and some other nations [14].

PUV irradiation is broad spectrum light with wavelengths ranging from ultraviolet to infrared (including UV-A, UV-B, UV-C, visible, and infrared wavelengths, spanning 200–1,100 nm) in which light-pulses are delivered at short durations (micro- to milliseconds) [13, 16, 17]. The high efficacy of pulsed-light is due to the higher amount of energy accumulation compared with continuous light that instantaneously discharges energy on its target. The energy distributed by the UV light source inactivates microorganisms by destroying DNA, thus providing a higher degree of decontamination, sanitation, and sterilization [18]. PUV is considered a nonthermal and nonchemical process when processing times are short; i.e., light energy is administered for a fraction of a second (milli- to microsecond) [17, 19]. Moreover, pulsed administration of UV-light is thousands of times more efficient at decontamination than continuous administration of UV-light [13, 17]. PUV light-induced inactivation of microorganisms occurs owing to a combination of photochemical, photothermal, and photophysical mechanisms [14, 16, 17, 20]. Photochemical inactivation resembles the inactivation mechanism of UV-C (200–280 nm) [11]. The photochemical effect alters the chemical structure of DNA by forming a thymine–thymine dimmer, preventing replication, and resulting in irreversible cellular injury and death [16, 20]. Depending on the food matrix, light penetration of microbial cells can result in vapors originating from cellular water sources. Osmotic imbalances can also occur owing to absorption that result in cytoplasmic shrinkage and cell rupture. Photophysical effects can cause direct damage to the cells causing leakage of cellular materials [11, 14, 16, 20]. PUV light has been shown to be an effective process for decontamination (of microbes or allergens) of many food products such as milk [13, 21], juice [22], spices [7], semi solid-foods such as liquid peanut butter [23], shrimps [17], shelled eggs [24], and meat [19, 25]. As a nonthermal post-harvest intervention method, PUV treatment is reported to be effective at reducing microbial loads on fruits and vegetables [26–28].

Researchers have shown that a low frequency pulsed light, UV-A light emitting diodes (UVA-LED), when administered to biofilms at 5- to 60-min pulses was more effective than 2.5- to 30-min UV exposure in continuous mode [29]. The PUV-mediated inactivation of microorganisms on small fruit surfaces has been reported [27]. Furthermore, the effectiveness of PUV at inactivating *Escherichia coli* [30], *Salmonella* [27], and *Listeria monocytogenes* [8] has been demonstrated. Previous studies have shown that PUV at low frequency is germicidal, and effective against harmful bacterial pathogens that are capable of forming biofilms [29]. However, to date, no studies have reported the effectiveness of PUV exposure on biofilms present on the surface of fresh produce and food packaging materials. In the current study, it is hypothesized that PUV will be effective in reducing surface contamination on fresh produce by reducing the numbers of viable cells in biofilms. To test this hypothesis, the effects of PUV process variables (such as time of exposure and distance from the strobe) were evaluated in the inactivation of biofilms formed by selected pathogens (*L. monocytogenes* and *E. coli* O157:H7) on a model leafy green produce (lettuce) and food contact system [low-density polyethylene (LDPE) packaging film].

## Results and discussion

### Formation of biofilms on test surfaces

**Figure 1:**
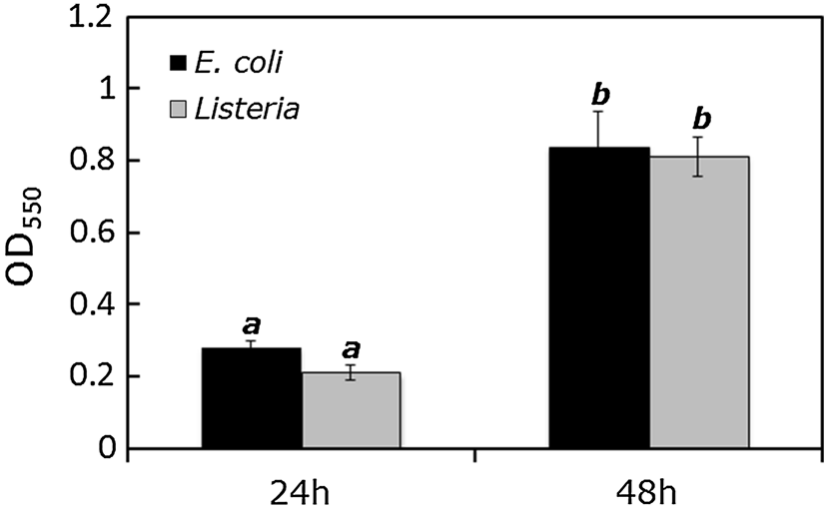
Microtiter plate-based in vitro biofilm formation assay of *E. coli* and *L. monocytogenes.* The formation of biofilm (at 24 and 48 h post-inoculation, at 30°C) was measured by optical density readings at 550 nm. Values are presented as the mean ± SE of three experiments, repeated eight times. *Columns* mean, *bars* SE. Columns with *different letters* indicate significant differences (*P* < 0.05).

The formation of biofilms on model surfaces (plastic petri dishes), Romaine lettuce, and packaging materials (LDPE bags) was evaluated qualitatively using crystal violet and Alcian blue staining methods, as described previously [31, 32]. The staining methods coupled with light microscopy provided direct evidence of biofilm formation by *E. coli* O157:H7 and *L. monocytogenes* on the test substrates mentioned above (data not shown). The results of in vitro microtiter plate-based biofilm formation assays of the two test pathogens at two different time points (24 and 48 h, at 30°C) are presented in Figure 1. At 48 h of incubation, the degree of biofilm formation was significantly higher (*P* < 0.05) than at 24 h incubation for both pathogens. The OD value for *E. coli* O157:H7 at 48 h was 0.84 ± 0.09, compared with 0.28 ± 0.02 at 24 h. For *L. monocytogenes*, the OD values at 24 and 48 h incubation (a measure of biofilm formation) were 0.21 ± 0.02 and 0.81 ± 0.05, respectively. It is apparent from the in vitro biofilm formation assays that the biofilm-forming microbial population increased over time at the test temperature, which is in agreement with several previous studies [31, 33–35].

### Microbial inactivation as a result of PUV light treatment

The inactivation of test microorganisms as a result of PUV-light treatment was evaluated by two different quantitative methods: plating on selective agar plates and an ATP luminescence-based assay.

#### Inactivation as enumerated by selective plating

**Figure 2:**
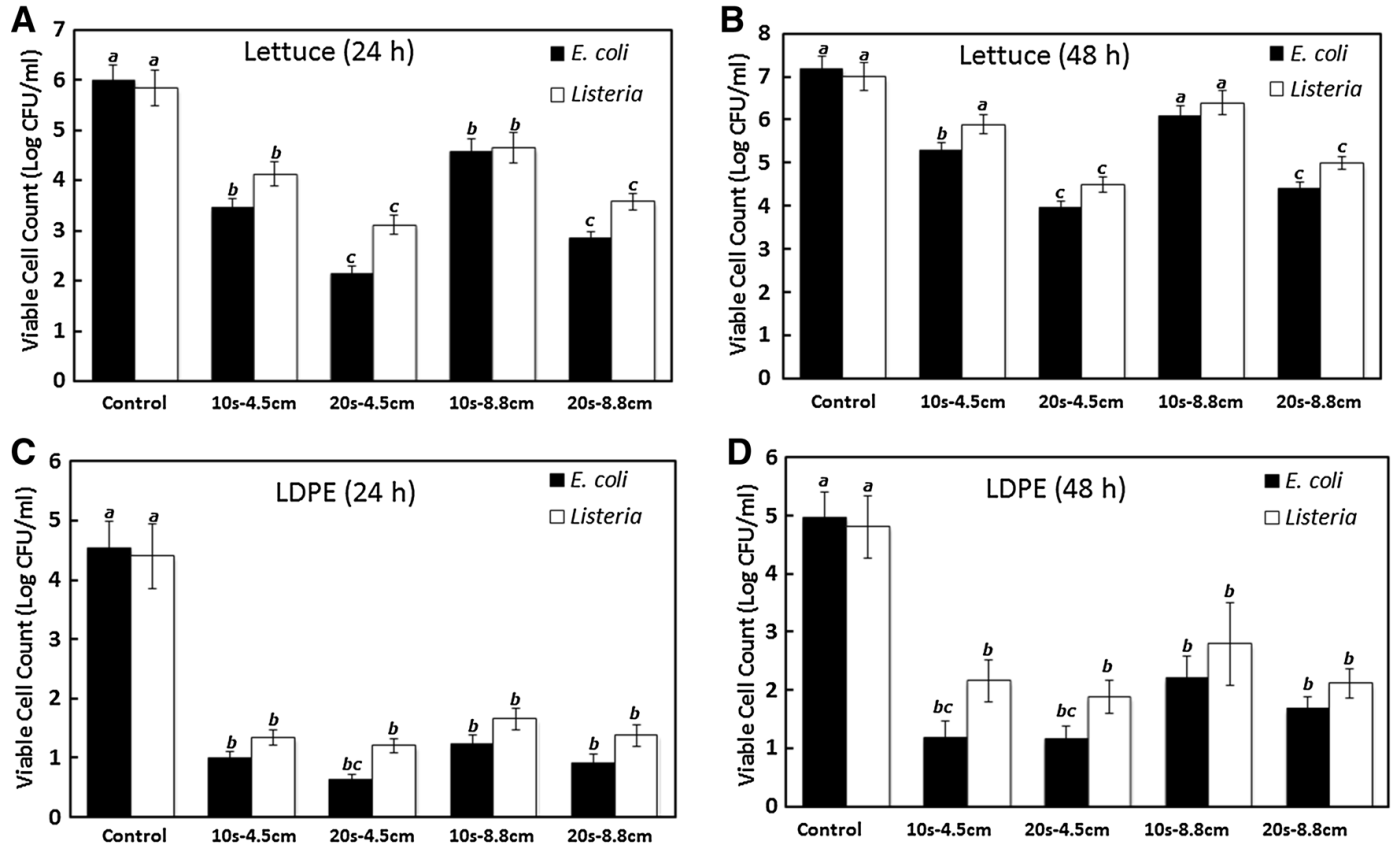
Survival of *E. coli* and *L. monocytogenes* in biofilms after PUV-light treatment. PUV-light treatment was performed under different exposure conditions, i.e., different times (in s) and distances (in cm) from the UV source to the samples. **A** Lettuce leaves incubated at 30°C for 24 h; **B** lettuce leaves incubated at 30°C for 48 h; **C** LDPE films incubated at 30°C for 24 h; **D** LDPE films incubated at 30°C for 48 h. Values are presented as the mean ± SE of two experiments performed in triplicate. *Columns* mean, *bars* SE.* Columns* labeled with *different letters* indicate significant differences (*P* < 0.05).

*E. coli* and *L. monocytogenes* cells in biofilm on the surfaces of lettuce and LDPE film pieces were treated with PUV-light at fluencies of 0.43 and 0.30 J cm^−2^ per pulse, which corresponded to 4.5 and 8.8 cm from the UV light source. The number of viable cells of *E. coli* and *L. monocytogenes* on lettuce biofilms (formed in 24 or 48 h) post-PUV treatment at different exposure times to sample distances was determined by selective plating, as depicted in Figure 2A, B. A longer PUV exposure time to shorter sample to UV light source distance (20 s—4.5 cm) resulted in a significant reduction in viable cell counts in biofilms formed by both of the test pathogens on lettuce leaves when compared with a shorter exposure time to longer light source distance (10 s—8.8 cm). PUV treatment of lettuce leaves (with 24-h *E. coli* biofilms) for 10 s at 4.5 and 8.8 cm distances from the light source resulted in a 2.5 log CFU mL^−1^ and 1.4 log CFU mL^−1^ reduction of viable cells, respectively, compared with the no treatment controls. Inactivation of the same 24-h *E. coli* biofilms on lettuce leaves led to a greater reduction (*P* < 0.05) in viable cells to 3.9 log CFU mL^−1^ (for 4.5 cm distance) and 3.1 log CFU mL^−1^ (for 8.8 cm distance) when the PUV exposure time was increased to 20 s (Figure 2A). The PUV-mediated reduction in viable counts for 48-h *E. coli* biofilms on lettuce leaves showed a similar trend, with the 10 s—4.5 cm and 20 s—4.5 cm treatments resulting in a reduction in viable cells of 1.9 log CFU mL^−1^ and 3.2 log CFU mL^−1^, respectively. For longer (8.8 cm) sample-UV light source distances, the reduction in viable cells was lessened to 1.1 log CFU mL^−1^ (for 10 s treatment) and 2.78 log CFU mL^−1^ (for 20 s treatment). In general, it was also observed that the biofilm formed by *E. coli* on lettuce leaves over a period of 48 h was more resistant to PUV light treatment compared with biofilms formed over 24 h (Figure 2A, B). Romaine leaf samples containing 24 or 48 h *L. monocytogenes* biofilms treated with PUV light for 20 s—4.5 cm showed significant (2.7- and 2.5-log CFU mL^−1^) reductions in viable cell counts compared with the no-PUV controls (Figure 2A, B) (*P* < 0.05). For a PUV light treatment of 10 s at a distance of 8.8 cm, the inactivation of *L. monocytogenes* biofilms resulted in reductions of viable cells of 1.19 log CFU mL^−1^ (for 24 h biofilms) and 0.6 log CFU mL^−1^ (for 48 h biofilms); these values were not significant when compared to PUV untreated controls (*P* > 0.05). Samples treated at 8.8 cm for 20 s, however, resulted in significantly reduced counts of viable *Listeria* cells and the inactivation of 2.25 and 2.01 log CFU mL^−1^ from the 24 and 48 h biofilms, respectively, compared with the control (no PUV) (*P* < 0.05). In all of the above cases, the extent of inactivation was calculated by subtracting the viable cell count of a particular treatment from the respective control value.

Pieces of LDPE film were used to mimic the food contact surface capable of harboring bacterial biofilms. These LDPE pieces were optically transparent and showed higher levels of PUV-light mediated inactivation compared with Romaine lettuce leaves. However, the overall pattern of inactivation of viable cells in biofilms for the test pathogens was similar to that seen with Romaine leaves. For the treatment of 10 s at a distance of 8.8 cm, the recorded inactivation values for *E. coli* O157:H7 were 3.29 and 2.76 log CFU mL^−1^ for the 24 and 48 h biofilms, respectively. When the treatment time was increased to 20 s (for 8.8 cm distance), the maximum *E. coli* O157:H7 inactivation was found to be 3.9 log CFU mL^−1^ (Figure 2C). *Listeria* biofilms offered more resistance to PUV-mediated inactivation on LDPE films compared with *E. coli* biofilms. For a PUV treatment of 10 s—8.8 cm, *Listeria* inactivation values were found to be 2.3 log CFU mL^−1^ (24 h) and 1.9 log CFU mL^−1^ (48 h). For *Listeria*, the maximum inactivation was found to be 2.8 log CFU mL^−1^ (PUV light treatment of 20 s—4.5 cm) (Figure 2C, D). Again, the inactivation values reported above were calculated by subtracting the viable cell counts after a particular treatment from the respective control values.

#### Inactivation as enumerated by a luminescence-based quantitative assay

The effect of PUV treatment on the viability of microorganisms forming biofilms was evaluated by measuring the ATP released from bacteria using a BacTiter-Glo™ Microbial Cell Viability Assay Kit (Promega). The results from this culture independent assay (Table 1) were used to confirm the results obtained by direct microbial plating. A PUV light treatment of 20 s—4.5 cm on *E. coli* O157:H7 biofilms on lettuce resulted in the lowest ATP bioluminescence values (i.e., lowest viability) for both of the time points resulting in approximately 1.2 log relative luminescence units (RLU) mL^−1^ (at 24 h) and 1.7 log RLU mL^−1^ (at 48 h). For all *E. coli* O157:H7 biofilms formed on lettuce leaves, PUV treatments showed significantly (*P* < 0.05) lower RLU values compared with the control (no PUV) (Table 1). However, for *L. monocytogenes* biofilms on lettuce, not all treatments showed significantly (*P* < 0.05) lower RLU values compared with the control (no PUV). For *Listeria* on lettuce, PUV treatment of 20 s—4.5 cm resulted in the highest inactivation yielding lower approximate ATP bioluminescence values of 2.5 and 2.8 log RLU mL^−1^ at 24 and 48 h, respectively. *Listeria* biofilms showed resistance to the PUV treatment of 10 s—8.8 cm, confirming the plating data, and indicating that the reduction in viable cells at this PUV light dosage was not significant (*P* > 0.05). For the PUV treatment of 10 s—8.8 cm, the luminescence values were around 3.9 log RLU mL^−1^ (for 24 h, the corresponding control value was ~4.8 log RLU mL^−1^) and 4.1 log RLU mL^−1^ (for 48 h, the corresponding control value was ~4.7 log RLU mL^−1^). For LDPE films, the PUV-mediated inactivation of both the test pathogens in biofilms was found to be significant (*P* < 0.05). For *E. coli* biofilms, the highest approximate ATP bioluminescence value was recorded as 1.65 log RLU mL^−1^ (PUV dosage of 10 s—8.8 cm at 48 h) while the lowest bioluminescence value was around 0.62 log RLU mL^−1^ (PUV dosage of 20 s—4.5 cm at 24 h). A similar trend was observed for *Listeria* biofilms; however, the RLU values were higher than the *E. coli* values for each corresponding treatment point. For example, a PUV dosage of 10 s—8.8 cm at 48 h yielded an approximate bioluminescence value of 2.8 log RLU mL^−1^ (highest for *Listeria*), and for PUV treatment of 20 s—4.5 cm at 24 h this value was 1.15 ± 0.2 log RLU mL^−1^ (lowest for *Listeria*). It is evident from these data that, upon PUV light treatment there was a higher population of surviving bacterial cells in *Listeria* biofilms than in *E. coli* biofilms.

**Table 1:** Viability of biofilm-encased *E. coli* and *L. monocytogenes* (on lettuce or LDPE) after PUV treatment

Microorganism	PUV-treatment [time (s)/distance (cm)]	Viable cell population (log RLU mL^−1^)			
		Lettuce		LDPE	
		24 h	48 h	24 h	48 h
*E. coli* O157:H7	Control (no PUV)	4.50 ± 0.34 A	4.40 ± 0.29 A	4.53 ± 0.35 A	4.04 ± 0.30 A
	10 s—4.5 cm	1.97 ± 0.22 C	2.35 ± 0.27 B	0.99 ± 0.17 B	1.34 ± 0.23 B
	20 s—4.5 cm	1.22 ± 0.20 C	1.74 ± 0.24 C	0.62 ± 0.15 C	1.20 ± 0.20 B
	10 s—8.8 cm	2.61 ± 0.31 B	2.65 ± 0.29 B	1.23 ± 0.24 B	1.65 ± 0.24 B
	20 s—8.8 cm	1.62 ± 0.25 C	2.04 ± 0.26 B	0.90 ± 0.16 B	1.38 ± 0.13 B
*L. monocytogenes*	Control (no PUV)	4.80 ± 0.35 A	4.70 ± 0.46 A	4.96 ± 0.32 A	4.71 ± 0.36 A
	10 s—4.5 cm	3.39 ± 0.24 B	3.78 ± 0.37 B	1.17 ± 0.22 C	2.15 ± 0.31 B
	20 s—4.5 cm	2.53 ± 0.22 C	2.88 ± 0.33 B	1.15 ± 0.20 C	1.88 ± 0.33 C
	10 s—8.8 cm	3.90 ± 0.30 AB	4.10 ± 0.40 A	2.20 ± 0.32 B	2.80 ± 0.41 B
	20 s—8.8 cm	2.83 ± 0.27 B	3.19 ± 0.36 B	1.67 ± 0.25 C	2.11 ± 0.36 B

PUV-light treatment was performed under four different exposure conditions, i.e., different times (in s) and distances (in cm). Each treatment was replicated twice and assays were performed in triplicate. Values (mean ± SE) are given for each surface (lettuce or LDPE) and each incubation time (24 or 48 h post-inoculation), and different letters denote significant differences (*P* < 0.05).

The results from this study demonstrate that PUV-light treatment has microbicidal effects on biofilms formed by two major foodborne pathogens, *E. coli* and *L. monocytogenes*. Another significant observation is that the PUV-treatment groups, as they relate to leaf or LDPE surfaces, exhibited differences in inactivation. The biofilm formed on the leaf surface showed less PUV-mediated inactivation compared with the biofilm formed on LDPE film (Table 1). This indicated that the surface on which the biofilm forms effects PUV-mediated inactivation. Obviously, the surface topographies and the composition of a lettuce leaf are quite different from that of LDPE film. Attachment of cells in so-called “shadows” or locations where PUV-light may not reach (like stomata or leaf cavities) could provide enhanced protection from interventions, as previously reported [36]. It is evident from the literature that the presence of organic materials may provide microbial cells with more resistance to UV-light mediated disinfection [37] or other chemical disinfectants [36], which may partially explain why bacterial cells attached to lettuce may exhibit higher resistance to PUV treatment. Moreover, PUV treatment is a light-mediated intervention; therefore, the optical transparency and reduced surface roughness of the packaging film may also effect inactivation, as previously reported [38].

In our experiments, *L. monocytogenes* cells in biofilms showed a relatively higher resistance to PUV-treatment compared with *E. coli* O157:H7 cells for both lettuce and LDPE surfaces. The enhanced resistance of biofilm-forming *Listeria* (compared with *E. coli*) to different antimicrobial treatments, including pulsed-light has been reported by several earlier studies [36, 37, 39–41]. Ölmez and Temur (2010) reported a higher reduction of *E. coli* than *Listeria* on lettuce leaves as a result of chlorine and organic acid treatments [36]. The higher inactivation of *E. coli* was also reported when dip wash treatment with organic acids was applied to iceberg lettuce [42]. The precise mechanism responsible for the differential PUV-light mediated inactivation of *E. coli* O157:H7 (strain EDL933) and *L. monocytogenes* (strain V7) remains to be determined. However, it is evident in the literature that the relative interaction between antimicrobials with bacterial cells (either in planktonic or sessile form) is complex [35] and depends on several factors, including the antimicrobial used [43–45], surface type [46], and the bacterial strain [47–49]. It has also been reported that the robustness of biofilms formed by Gram-positive and Gram-negative bacteria may be attributed to cell wall structure, secreted compounds, and growth factors [50]. In Gram-positive bacteria (such as *Listeria*) the peptidoglycan layer is thick compared with Gram-negative bacteria, this differential thickness in peptidoglycan may contribute to differences in PUV-mediated inactivation, as proposed previously [51]. Moreover, a thick peptidoglycan layer consists of more sugars and amino acids and secreted residues, potentially aiding the formation of a firmer biofilm [50, 52]. However, no definite mechanistic explanation has been proposed to date for why PUV-treatment was less effective on biofilms formed by *L. monocytogenes* than *E. coli* O157:H7.

### Temperature profiles of PUV light treatment

**Figure 3:**
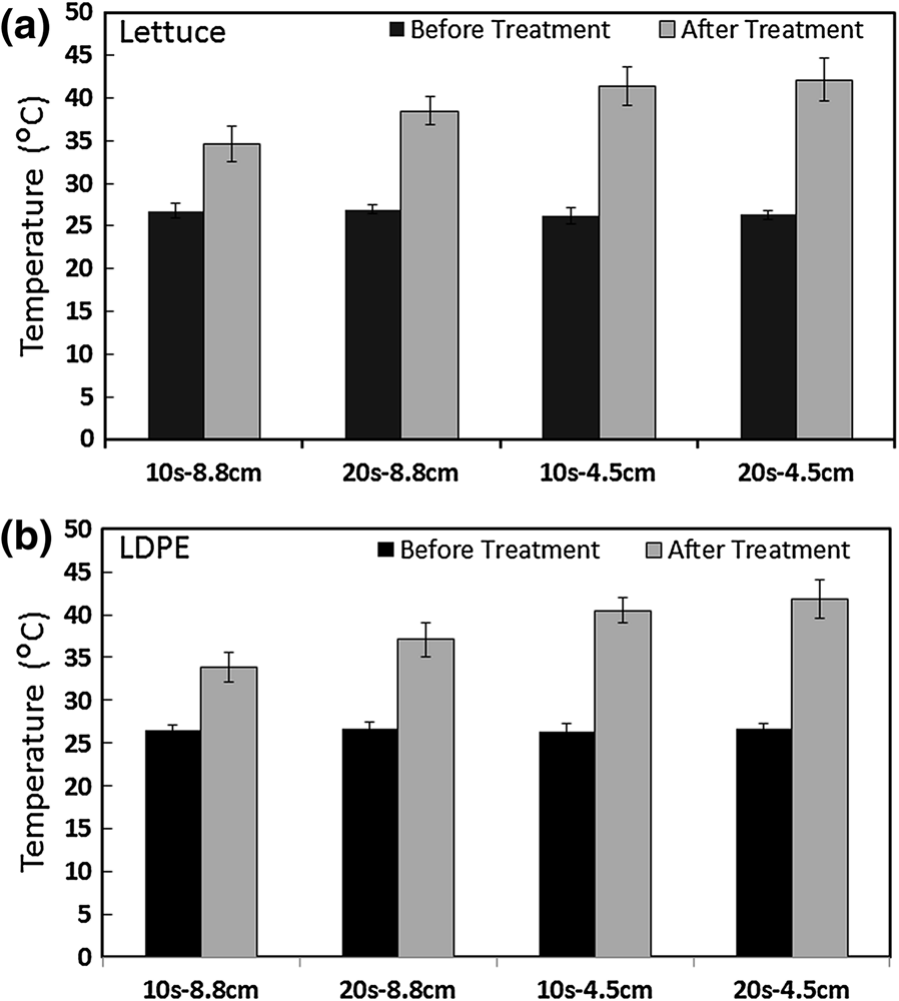
Surface temperature profile of lettuce and LDPE films after PUV treatment. The surface temperatures of lettuce leaves (**a**) and LDPE films (**b**) were measured at distances of 8.8 and 4.5 cm from the UV light source, before and after 20 or 10 s exposures. Values are presented as the mean ± SE of two experiments performed in triplicate. *Columns* mean, *bars* SE.

To assess the extent of heat generated by the PUV process, surface temperatures of the samples were recorded using an infrared thermometer. The temperatures at sample distances of 8.8 and 4.5 cm from the UV light source are given in Figure 3. The maximum surface temperature increase of 15.8 ± 2.6°C was observed for a PUV dosage of 20 s—4.5 cm on lettuce, with a highest recorded temperature post-treatment of 42.1 ± 2.5°C. When the PUV light treatment was administered for 10 s at a distance of 8.8 cm, the highest surface temperature was found to be 34.6 ± 2.1°C (for lettuce), and 33.9 ± 1.7°C (for LDPE). These temperature data indicated that the PUV process resulted in some instant heat generation. It was also observed that with a longer exposure time and shorter treatment distance (20 s—4.5 cm) more heat was generated (as measured by the temperature data) than with a shorter exposure time and longer treatment distance (10 s—8.8 cm). Overall, the temperature increase as a result of PUV treatment was in the range of ~7.4–15.8°C across all the treatment conditions tested. The temperature data collected in the current study are well within the range of several previously reported studies [8, 38, 53–58]. The results of the current study, in conjunction with the previous studies mentioned above, indicate that the increase in temperature resulting from PUV treatment is dependent on several factors, such as distance from the UV source to the target sample, frequency and duration of pulses, energy levels or fluences, and food or target surface type. The results from previous studies indicated that a UV-source-to-sample distance of approximately 10 cm may be used to avoid excessive heating during PUV light treatments [38, 56, 59].

### PUV light mediated damage of bacterial cells in biofilms

**Figure 4:**
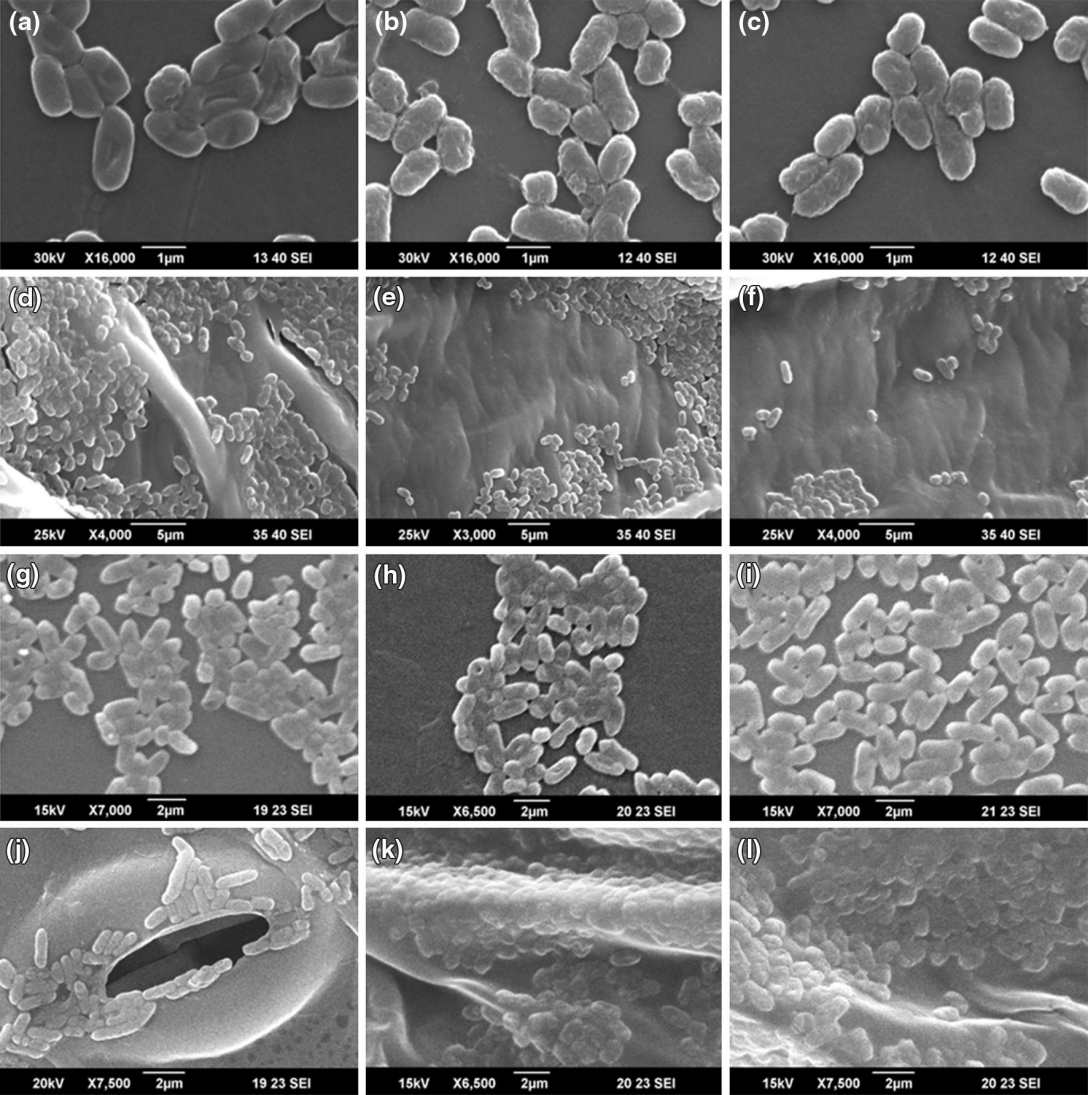
Scanning electron micrographs of pre- and post-PUV-treated *E. coli* and *L. monocytogenes* cells in biofilms. The micrographs are representative of cells in biofilms at 48 h post-inoculation. The images represent cells in biofilms receiving no PUV treatment (control), or PUV treatment for 20 s at a sample to light source distance of 4.5 and 8.8 cm (treated). **a** *E. coli* cells on plastic (control); **b** and **c** *E. coli* cells on plastic (treated) 8.8 cm (**b**) and 4.5 cm (**c**); **d** *E. coli* cells on lettuce (control); **d** and **e** *E. coli* cells on lettuce (treated) 8.8 cm (**e**) and 4.5 cm (**f**); **g** *L. monocytogenes* cells on plastic (control); **h** and **i** *L. monocytogenes* cells on plastic (treated) 8.8 cm (**h**) and 4.5 cm (**i**); **j** *L. monocytogenes* cells on lettuce (control); **k** and **l** *L. monocytogenes* cells on lettuce (treated) 8.8 cm (**k**) and 4.5 cm (**l**).

**Figure 5:**
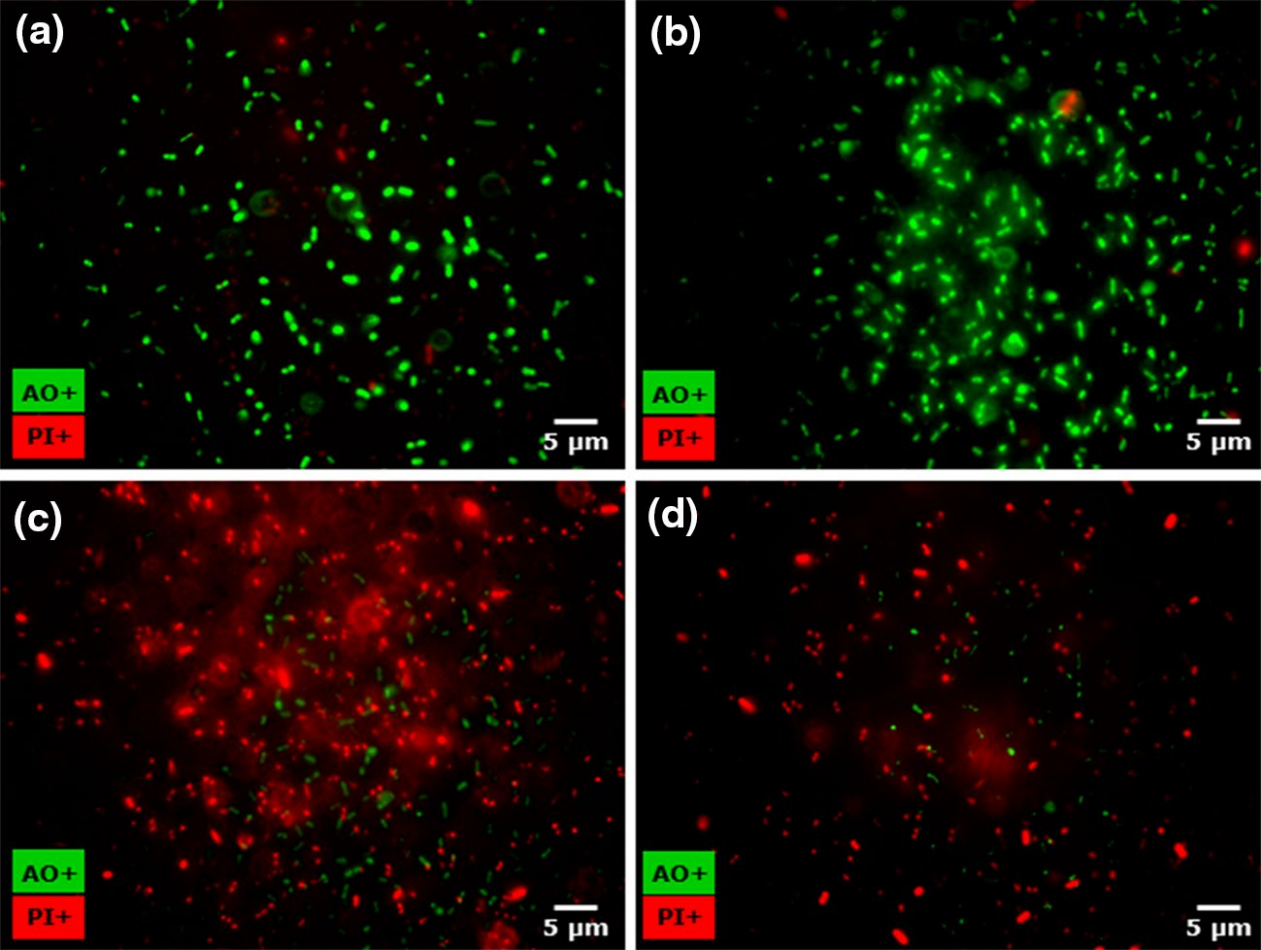
Fluorescence images of pre- and post-PUV-treated *E. coli* and *L. monocytogenes* cells in biofilms. The images represent cells in biofilms (48 h post-inoculation) receiving no PUV treatment (control), or PUV treatment for 20 s at a sample to light source distance of 8.8 cm (treated). The *upper panels* represent control cells of *L. monocytogenes* (**a**) and *E. coli* (**b**) recovered from lettuce. *Lower panels* depict the viability status of cells post-PUV treatment, *L. monocytogenes* (**c**) and *E. coli* (**d**) recovered from lettuce. Live or viable cells are represented by *green* fluorescence (AO+), while *red* fluorescence (PI+) indicates dead cells.

The fate of *E. coli* O157:H7 and *L. monocytogenes* cells on lettuce and LDPE surfaces under the experimental conditions (PUV treatment, 20 s—8.8 cm and 20 s—4.5 cm) were evaluated by scanning electron microscopy (SEM) (Figure 4a–l). The formation of biofilm-like structures (i.e., extracellular polymeric substance-mediated aggregate formation) by both pathogens on lettuce and LDPE surfaces was evident at 48 h post-inoculation, but not at 24 h. Niemira and Cooke (2010) reported similar findings of the time-dependent formation of biofilm-like structures of *E. coli* on lettuce and spinach leaves [33]. The micrographs of control cells at 48 h post-inoculation also depicted cell crowding (Figure 4d–l), which is also indicative of nascent biofilm formation, as reported previously [33, 36]. At the individual cell level, minor morphological changes were evident in PUV-treated bacterial cells compared with control cells (Figure 4a–l). The PUV-treated cells appeared to have increased roughness on their surfaces, showing some signs of shrinkage (Figure 4b, c, e, f, h, i, k, l). In a recent study, Ramos-Villarroel et al. (2012) reported significant damage of the PUV-treated cell membrane in microorganisms [51]. Alterations in the bacterial cell membrane resulting from PUV treatment were also reported in another recent study [60]. The initial electron micrograph findings from the current study may indicate possible alterations or damage of the bacterial cell membrane structure as a result of PUV treatment, confirming the findings of others [51, 61]. However, this morphological change, which may indicate alteration or damage of the bacterial cell wall and cell membrane structures, should be interpreted with care and may not solely be attributable to PUV, rather it may be a contributory factor along with DNA structural damage (thiamine dimer formation) contributing to cell injury and death [10]. To supplement our findings of electron microscopy experiments by fluorescence microscopy method, we recovered a subset of PUV treated cells (20 s—8.8 cm) from biofilms formed on lettuce leaf surfaces over a period of 48 h. The extent of bacterial inactivation as a result of PUV treatment was evaluated by intake of fluorescence dyes, acridine orange (AO, green) and propidium iodide (PI, red). Figure 5 depicts fluorescence micrograph images of untreated (no PUV treatment) and PUV treated (20 s—8.8 cm) cells from lettuce. Visual observations reveal a significant higher number of dead (red) bacterial cells in PUV treatment group (Figure 5c, d) as compared to the control group (Figure 5a, b). It is evident that in PUV treated cells, the live cell population is higher for *L. monocytogenes* (Figure 5c) than *E. coli* O157:H7 (Figure 5d). The findings of fluorescence microscopy also confirms our selective plating and luminescence-based quantitative assay results indicating enhanced resistance of biofilm-forming *Listeria* (compared with *E. coli*) to PUV treatment.

## Conclusions

The results from the current study indicate a moderate inhibitory effect of PUV treatment on LDPE food packaging material and Romaine lettuce surfaces harboring viable *E. coli* O157:H7 and *L. monocytogenes* cells in nascent biofilms. The PUV-mediated microbial inactivation values were found to range from approximately 0.6 log CFU mL^−1^ to 4 log CFU mL^−1^. Microbial inactivation due to PUV treatment was found to be dependent on several factors, including the PUV-treatment dosage, the microorganism type, and the type of material supporting the biofilm. In general, biofilms formed on the leaf surface showed less PUV-mediated inactivation compared with biofilms formed on LDPE film. The process generated nonsignificant amounts of heat, and can be considered as a nonthermal intervention method for reducing the microbial load in biofilms. This study provides preliminary evidence that PUV treatment can reduce the microbial load on produce and food packaging material surfaces, and therefore, in the future, this process may effectively be employed for surface decontamination of leafy produce and food contact areas.

## Methods

### Bacterial cultures, media, and growth conditions

*E. coli* O157:H7 strain EDL933 (ATCC 43895) and *L. monocytogenes* strain V7 (½ a) were obtained from the Food Microbiology Culture Collection at Alabama A&M University. Both of these strains are associated with several foodborne outbreaks [62–64]. They are also known to produce firm biofilm structures, and contain all major virulence related genes [65, 66]. The stock cultures were kept at −80°C in 15% (vol/vol) glycerol for long-term storage. For routine propagation, cultures from the frozen stock were transferred to 10 mL of sterile brain–heart infusion (BHI) broth (Becton–Dickinson, Sparks, MD, USA), using a 0.1% (vol/vol) inoculum, and incubated at 37°C overnight (referred hereafter as ON culture) prior to experimentation. The bacterial concentrations in the broth were adjusted by optical density (OD at 600 nm, OD_600_), followed by plating and enumeration at appropriate dilutions. For biofilm assays, ON cultures were washed three times in PBS, diluted, and incubated at 30°C for different durations (h) [67]. For selective enumerations of *E. coli* O157:H7 and *L. monocytogenes*, sorbitol MacConkey agar supplemented with cefixime and tellurite, and modified Oxford agar were used, respectively (Becton–Dickinson).

### Biofilm formation in vitro

One hundred microliters of the diluted (1:100) ON culture suspensions (in BHI) were added to polystyrene 96-well microplates (Corning, Lake Placid, NY, USA) incubated under static conditions at 30°C for 24 and 48 h. Eight replicate wells for each microorganism were inoculated. After incubation, the excess medium was removed from the wells and one plate was used for confirmation of attachment and formation of biofilm using previously described crystal violet staining methods [31, 68] with some modifications. Briefly, plates were washed five times with PBS, then placed in a biosafety cabinet (BSC) for air drying (45 min) and 125 μL of 0.1% solution of crystal violet in water was added to each well and incubated for 45 min at room temperature. Crystal violet was removed, and the plate was washed five times with PBS. After this step, the plates were air dried for 45 min inside a BSC laminar flow hood with continuous air circulation. Then, 125 µl of 30% acetic acid in water was added to each well of the plate and mixed to dissolve the crystal violet dye. Measurement of biofilm formation in each well was performed by recording the OD at 550 nm (OD_550_) using 30% acetic acid in water as the blank. Alcian blue staining of biofilm-associated acidic polysaccharides (a major constituent of extracellular polymeric substances) was also performed, using a previously reported method [32], to confirm the crystal violet data.

### Preparation of produce and model food contact surface prototypes

Whole heads of Romaine lettuce were purchased from a local supermarket 1–2 days after stocking, and were used on the same day. To maintain the uniformity among different samples purchased on different days, the brand of the product and the supermarket remained unchanged during the study. For each head of lettuce, the two outermost leaf layers were discarded. The inner leaves were aseptically removed, and were cut into 3 × 3 cm pieces using sterile scissors. All pieces were stored in empty 100 × 15 mm petri dishes (Fisher Scientific, Pittsburgh, PA, USA) with water-soaked Kimwipes® (sterilized) to preserve humidity. To generate a prototype food contact surface (to represent packaging), LDPE bags (2 MIL; Uline, Waukegan, MI, USA) were used. The LDPE bags were aseptically cut into 3 × 3 cm pieces of film. The cut pieces were sterilized by wiping with 70% ethanol followed by air drying in a BSC and were used for inoculation.

### Inoculation of lettuce leaves and packaging films and recovery of attached cells

The pieces of lettuce or packaging (LDPE) films were inoculated with *E. coli* O157:H7 or *L. monocytogenes.* The pieces were submerged in the bacterial suspension (10^4^ CFU mL^−1^ in BHI) in the wells of sterile 24-well plates (1 piece per well in 400 µl suspension). Un-inoculated pieces were included in the study to verify the absence of target pathogens. The petri dish containing the leaf or LDPE film pieces were incubated at 30°C for 24 and 48 h. The formation of biofilms was confirmed by the crystal violet staining method (as described in the earlier section) and by SEM (described in a later section). One replicate was dipped in 10 mL of saline for 3 s to remove residue. The physical detachment of bacteria from produce (or LDPE film) was conducted by a previously described method [31]. Each piece of lettuce leaf or LDPE film was transferred to a 50 mL tube containing 25 mL of PBS and vortexed for 20 s to remove loosely attached bacteria. To recover strongly bound cells, the same tubes were sonicated for 30 s at 50% power using a Fisher Scientific Sonic Dismembrator (Model 50), and 200 µl of the supernatant was diluted and plated on selective agar. Between each sonication, the sonicator probe was sterilized with 70% ethanol and rinsed with distilled water. For enumeration, selective agar plates were incubated for 24 h at 37°C.

### Treatment with PUV light

A laboratory-scale pulsed-light system (SteriPulse-XL 3000, Xenon Corp., Wilmington, MA, USA) was used to administer PUV light. A detailed description and operating details of the PUV system can be found at the manufacturer’s (Xenon) website and in previously published reports [12, 21, 53]. According to the manufacturer’s specifications, the pulsed light system generated 1.27 J cm^−2^ per pulse of broadband energy (200–1,100 nm) at 7.6 cm below the central axis of the pulsed UV lamp. The energy distributions were approximately 54, 26, and 20% in the ultraviolet, visible, and infrared regions, respectively. The system produced three pulses per second with a width of 360 μs for an input of 3,800 V [23, 27, 69]. The pieces of lettuce and LDPE films were treated at two distances (4.5 and 8.8 cm) from the UV light source, and were exposed to PUV light for two time durations (10 and 20 s). Before and immediately after (within 5 s or less) each treatment, the surface temperature on the sample pieces was measured using an infrared thermometer (Fisher Scientific) [38].

### Microbiological analyses

#### Selective plating method

After treatment, the attached cells from the samples (pieces of leaves or LDPE films) were recovered as described above. A 100 µl aliquot of the appropriate dilution (in duplicate) of the vortexed and sonicated solution was plated on sorbitol MacConkey agar supplemented with cefixime and tellurite or modified Oxford agar (in triplicate for each dilution) for enumeration. The plates were incubated for 24 h at 37°C. Appropriate controls were established by plating non-PUV treated lettuce leaves or LDPE film samples.

#### Quantitative assay of microbial viability

The effect of PUV treatment on the viability of the biofilm-forming microorganisms was evaluated using a BacTiter-Glo™ Microbial Cell Viability Assay Kit (Promega, Madison, WI, USA) according to the manufacturer’s instructions. Briefly, an aliquot of 100 µl of bacterial cell suspension (dislodged from the leaf or LDPE surface) after PUV treatment of the bacterial samples were transferred to 96-well microtiter plates. An equal volume (100 µl) of BacTiter-Glo™ reagent was then added to the wells, the plates were mixed briefly on a shaker, and the luminescence was recorded using a microplate luminometer (BioTek Synergy HT, Winooski, VT, USA) for the quantitative enumeration of the live-dead status of bacterial cells upon PUV treatment. The data were expressed as RLU. ATP solutions of concentrations ranging from 0.1 to 100 nM were used as internal controls to standardize all experiments. The RLU values of the supernatant of vortexed and sonicated lettuce leaves and LDPE piece samples (non-inoculated) were used to normalize inoculated sample RLU values.

#### Fluorescence microscopy analysis

Bacterial cell death as a result of PUV exposures were determined by using a fluorescence microscopy method described previously [70]. Briefly, a cell staining solution containing 20 μg/mL of acridine orange (AO) and 100 μg/mL propidium iodide (PI) (Sigma) were prepared in PBS. The bacterial cells (exposed to PUV, as described in previous sections) were dislodged from leaf surfaces by sonication and vortexing. A 100 μL aliquots of cell suspension was mixed with 100 μL of staining solution and analyzed immediately with a fluorescence microscope (Nikon Eclipse TS 100, with SPOT software, version 4.6.4.2, Diagnostic Instruments, Sterling Heights, MI, USA) using green (for AO) and red filters (for PI). The detection of live (L) and dead (D) cells were conducted in the following manner, green (L) and red (D), both by visual scoring on a fixed microscopic field and by using image analysis software, SPOT, version 4.6.4.2 (images acquisition) and ImageJ v1.38 (NIH, USA) with “color counter” (v2001) and “color histogram” plug-ins (v2007) to analyze and enumerate the images.

### Scanning electron microscopic analysis

For SEM analyses, the inoculated lettuce leaves and LDPE films were processed, mounted, and sputter-coated following a method reported by Niemira and Cooke [33]. The SEM images were acquired using a JEOL 6390 LV electron microscope (JOEL, Tokyo, Japan) operating at voltages of 15–30 kV in the high vacuum mode. The surfaces of the test materials (lettuce leaves and LDPE) were examined for the confirmation of typical sessile forms of bacterial aggregates with respect to the formation of biofilms, for both control and PUV-treatments at each post-inoculation time point. Two independent trials of SEM experiments (including sample preparations and imaging) were conducted.

### Statistical analysis

The effect of PUV parameters (time and distance) on microbial destruction was investigated by two independent trials (performed in triplicate). Surviving microbial counts (viable count) were converted to log CFU mL^−1^. For each data point, the standard error of the mean (SE) was estimated, and data were expressed as the mean ± SE (error bars in figures indicate SE estimates). The data for microbial counts were subjected to analysis of variance (ANOVA) and Tukey’s test using SAS software (version 9.3, SAS Institute, Cary, NC, USA) for comparisons of microbial inhibition values between control and PUV-exposed sample means. The limit for statistical significance was set at *P* < 0.05.
